# Molecular Mechanisms Responsible for Diabetogenic Effects of COVID-19 Infection—Induction of Autoimmune Dysregulation and Metabolic Disturbances

**DOI:** 10.3390/ijms241411576

**Published:** 2023-07-18

**Authors:** Barbara Grubišić, Luka Švitek, Klara Ormanac, Dea Sabo, Ivica Mihaljević, Ines Bilić-Ćurčić, Tea Omanović Kolarić

**Affiliations:** 1 Department of Infectious Diseases, University Hospital Centre Osijek, 4 Josip Huttler Street, HR-31000 Osijek, Croatia; barbara.grubisic@kbco.hr (B.G.); luka.svitek@kbco.hr (L.Š.); 2 Faculty of Medicine Osijek, J. J. Strossmayer University of Osijek, 4 Josip Huttler Street, HR-31000 Osijek, Croatia; 3 Department of Pharmacology, Faculty of Medicine Osijek, J. J. Strossmayer University of Osijek, 4 Josip Huttler Street, HR-31000 Osijek, Croatia; klara.ormanac@mefos.hr (K.O.); dea.sabo@mefos.hr (D.S.); tomanovic@fdmz.hr (T.O.K.); 4 Clinical Institute of Nuclear Medicine and Radiation Protection, University Hospital Centre Osijek, 4 Josip Huttler Street, HR-31000 Osijek, Croatia; ivica.mihaljevic@mefos.hr; 5 Department for Nuclear Medicine and Oncology, Faculty of Medicine, J. J. Strossmayer University of Osijek, 4 Josip Huttler Street, HR-31000 Osijek, Croatia; 6 Academy of Medical Sciences of Croatia, 15 Kaptol Street, HR-10000 Zagreb, Croatia; 7 Department of Endocrinology and Metabolism Disorders, Internal Medicine Clinic, University Hospital Centre Osijek, 4 Josip Huttler Street, HR-31000 Osijek, Croatia; 8 Faculty of Dental Medicine and Health Osijek, University of Osijek, 21 Crkvena Street, HR-31000 Osijek, Croatia

**Keywords:** COVID-19, SARS-CoV-2, diabetes, autoimmune dysregulation, new-onset diabetes, hyperglycaemia insulin resistance

## Abstract

The COVID-19 pandemic has revealed a significant association between SARS-CoV-2 infection and diabetes, whereby individuals with diabetes are more susceptible to severe disease and higher mortality rates. Interestingly, recent findings suggest a reciprocal relationship between COVID-19 and diabetes, wherein COVID-19 may contribute to developing new-onset diabetes and worsen existing metabolic abnormalities. This narrative review aims to shed light on the intricate molecular mechanisms underlying the diabetogenic effects of COVID-19. Specifically, the review explores the potential role of various factors, including direct damage to β-cells, insulin resistance triggered by systemic inflammation, and disturbances in hormonal regulation, aiming to enhance our understanding of the COVID-19 impact on the development and progression of diabetes. By analysing these mechanisms, the aim is to enhance our understanding of the impact of COVID-19 on the development and progression of diabetes. The binding of SARS-CoV-2 to angiotensin-converting enzyme 2 (ACE2) receptors, which are present in key metabolic organs and tissues, may interfere with glucometabolic pathways, leading to hyperglycaemia, and potentially contribute to the development of new disease mechanisms. The virus’s impact on β-cells through direct invasion or systemic inflammation may induce insulin resistance and disrupt glucose homeostasis. Furthermore, glucocorticoids, commonly used to treat COVID-19, may exacerbate hyperglycaemia and insulin resistance, potentially contributing to new-onset diabetes. The long-term effects of COVID-19 on glucose metabolism are still unknown, necessitating further research into the possibility of developing a novel type of diabetes. This article provides a comprehensive overview of the current understanding of the interaction between COVID-19 and diabetes, highlighting potential areas for future research and therapeutic interventions.

## 1. Introduction

During the COVID-19 pandemic, it has been observed that individuals with diabetes have a significantly increased risk of developing a severe form of the illness and a higher mortality rate following infection with SARS-CoV-2 [1]. This is consistent with the long-established connection between diabetes and increased susceptibility and severity of infections, which is attributed to hyperglycaemia. Hyperglycaemia leads to cytokine dysregulation and immune response alteration, resulting in a pro-inflammatory and procoagulant state that promotes immune dysfunction through various pathways [2,3]. Individuals with diabetes also exhibit increased rates of hospitalization and mortality resulting from infections. The risk of infections escalates with worsening glycaemic control, with type 1 diabetes patients being at greater risk [4,5,6]. Individuals diagnosed with type 1 diabetes mellitus (T1DM) or type 2 diabetes mellitus (T2DM) frequently present with comorbid medical conditions, such as hypertension, obesity, and cardiovascular disease, which have been linked to an increased risk of contracting COVID-19 as well as an increased risk of infection-related mortality [7]. As the COVID-19 pandemic is constantly evolving, it has become more apparent that individuals with COVID-19 may experience hyperglycaemia, regardless of whether they have diabetes. This observation could imply a mutually influential relationship between COVID-19 and diabetes. Recent research suggests that a combination of insulin resistance and possible issues with insulin secretion may be responsible for the development of hyperglycaemia in COVID-19 patients who did not previously have diabetes [8,9].

The SARS-CoV-2 virus has the ability to attach itself to receptors called angiotensin-converting enzyme 2 (ACE2), which are present in various crucial metabolic organs and tissues, such as pancreatic β-cells, adipose tissue, kidneys, and the small intestine. This suggests that SARS-CoV-2 may potentially interfere with the glucometabolic pathways, leading to complications and even contributing to the development of new disease mechanisms [1]. Various mechanisms have been suggested to explain the occurrence of diabetes in conjunction with COVID-19 infection, including direct invasion of β-cells by the virus, leading to their impaired function, induction of insulin resistance through systemic inflammation, or endocrine alterations inciting this response [10]. It is presently uncertain if the emergence of SARS-CoV-2-induced diabetes is due to established mechanisms of type 1 diabetes mellitus (T1DM) or type 2 diabetes mellitus (T2D) or if it constitutes an atypical diabetes form. It is unknown whether COVID-19 patients are still vulnerable to developing new-onset diabetes or diabetes-related complications even after virus clearance and recovery [2,3]. Furthermore, glucocorticoids, which are frequently prescribed for moderate to severe COVID-19 cases, have been known to cause hyperglycaemia and insulin resistance, which could contribute to the incidence of new-onset diabetes [11]. Some studies have found that COVID-19 patients who develop new-onset diabetes tend to have worse outcomes than those with no diabetes or with previous diabetes [11,12,13,14]. Although the underlying mechanisms between COVID-19 and diabetes are still being investigated, it is obvious that both conditions share stress-induced pathways that interact in a two-way direction. This review aims to explore the available literature on SARS-CoV-2-related new-onset diabetes and the underlying physiological mechanisms.

## 2. Viruses and Diabetes Mellitus—What Do We Know So Far?

There is a great deal of speculation about viruses causing or triggering different types of chronic diseases, such as diabetes mellitus. This association was examined in studies with different viruses. For example, it was shown that enteroviruses can cause induction or acceleration of the autoimmune response to the insulin-producing β-cells of the pancreas [15,16,17]. Another example is the hepatitis C virus, which has been linked to an increased possibility of developing diabetes in liver transplant and other patients [18,19,20,21,22,23]. Furthermore, there was a large TEDDY study with 7896 participants and two relatively smaller studies—the MIDIA study with 885 participants and the BABYDIET study with 148 participants—that all confirmed an association between respiratory infection and diabetes development [24,25,26]. Al-Sayyar et al. focused on similarities between COVID-19-associated diabetes and other respiratory infections associated with diabetes, demonstrating common mechanisms among those groups of patients [27]. Because COVID-19 has been a major cause of respiratory infections since its outbreak in the year 2019, and as a relatively newly emerged disease, it presents an area of particular interest among scientists.

## 3. COVID-19 and Diabetes Mellitus

Clinical studies worldwide indicate that diabetes mellitus is among the most prevalent comorbidities observed in patients infected with SARS-CoV-2. At the onset of the COVID-19 pandemic, this finding, in association with the established heightened risk of infection with other pathogens, gave rise to the notion that individuals with diabetes are at a greater primary risk of contracting COVID-19. However, most of the studies reporting this finding pertain to patients receiving in-hospital care or those admitted to the intensive care unit (ICU), who typically experience a more severe course of the disease [28]. Several factors present in diabetes mellitus make individuals more susceptible and increase the severity of COVID-19 infection, including older age, a pro-inflammatory and procoagulable state, hyperglycaemia, and accompanying comorbidities like obesity, hypertension, cardiovascular disease, and chronic kidney disease. The state of hyperglycaemia, insulin resistance, and chronic low-grade inflammation leads to a dysfunction of the immune system, resulting in reduced interleukin production, decreased chemotaxis and phagocyte activity, and immobilization of polymorphonuclear leukocytes [28,29,30].

SARS-CoV-2 uses the ACE2 receptor to enter cells within the human body. People with diabetes mellitus have a greater level of expression of ACE2, which is primarily found in the lungs but also present in numerous other tissues, such as the endothelial cells of the heart and kidneys, as well as β-cells of the pancreas. Research has indicated that SARS-CoV-2 can replicate within the β-cells of the pancreas, causing harm to the pancreas and ultimately leading to hyperglycaemia and diabetic ketoacidosis [31]. The aforementioned mechanism could explain the increased susceptibility of patients with DM to illness caused by the COVID-19 infection and the emergence of more severe forms of the disease [30,32]. Both the viral spike protein binding to the ACE2 receptor and the degree of the immune response to the virus can be affected by uncontrolled hyperglycaemia. Elevated levels of blood sugar can directly increase the concentration of glucose in airway secretions [33]. Brufsky suggests that uncontrolled hyperglycaemia may increase in ACE2 receptors that are highly and aberrantly glycosylated in the lung, nasal airways, and oropharynx. This could lead to more binding sites for the SARS-CoV-2 virus, increasing the likelihood of COVID-19 infection and the severity of the disease [34]. This observation is suggestive of stress hyperglycaemia, which exhibits worse outcomes in acute illness when compared to pre-existing diabetes [35]. Stress hyperglycaemia has been identified as an unfavourable prognostic factor and has been associated with an elevated risk of respiratory failure and mortality in individuals with SARS [36]. The viral and immune responses during critical stages of COVID-19 can also lead to hyperglycaemia and reduce insulin sensitivity, resulting in additional metabolic complications [37]. Additionally, prolonged hyperglycaemia can impede the innate and humoral immune responses, thereby blocking lymphocyte proliferation, natural killer cell activity, and the function of monocytes/macrophages and neutrophils [38]. Correspondingly, numerous reports have demonstrated that elevated glucose levels upon admission serve as independent risk factors for the critical progression of COVID-19 and mortality [29,39,40].

SARS-CoV-2 directly affects the vascular system by targeting endothelial cells, resulting in severe endothelial damage and inflammation [41]. Additionally, COVID-19 causes an overproduction of pro-inflammatory cytokines, which further promotes endothelial dysfunction, which is already compromised by diabetes [42]. Three post-mortem histological analyses of patients have shown evidence of endotheliitis during COVID-19 infection, highlighting the potential for more severe clinical presentation in patients with a history of endothelial dysfunction [43]. Further evidence suggests that persistent endothelial dysfunction increases the susceptibility to severe COVID-19 disease. This is because hyperglycaemia and insulin resistance result in endothelial dysfunction and glycocalyx damage in individuals with type 2 diabetes mellitus, which then leads to leukocyte adhesion and promotes a procoagulant and antifibrinolytic state [44]. The combination of glucotoxicity and the inflammatory cytokine cascade characteristic of COVID-19, as well as immune dysregulation and endothelial damage, can result in additional metabolic complications in people with diabetes, including heightened susceptibility to thromboembolic events and multiorgan damage [45].

Thrombotic complications play a substantial role in the reduced survival rates observed in COVID -19 patients. Various studies have reported that the incidence of symptomatic venous thromboembolic events in individuals with COVID-19 ranges from 20% to 3% [46]. In contrast, the incidence of arterial thrombosis appears to be significantly lower than that of venous thromboembolism, with reported rates between 2.8% and 3.8% in small series [47]. Numerous studies indicate that COVID-19 infection can cause both arterial and venous thrombosis. Two distinct patterns of thrombotic manifestations have been identified, with one resembling classical thromboembolic disease and the other being diffuse micro-thrombotic [48]. Violi et al.’s research revealed that hospitalised COVID-19 patients are vulnerable to both venous and arterial ischemic events, which are indicators of a poor prognosis. This study contradicts previous findings by showing that COVID-19 is associated with a comparable venous and arterial thrombosis incidence. Nearly half of the 75 patients with ischemic events were affected by arterial thrombosis, altering coronary, cerebral, and peripheral circulations [49]. This thrombotic state is caused by the interaction between the inflammatory and hemostatic systems, including infected endothelial cells, leukocytes, platelets, complement activation, and the virus-induced hypoxic environment [48].

The association between COVID-19 and lipid metabolism has been observed, with liver damage being suggested as a potential etiological factor. Approximately half of COVID-19 patients exhibit mild to moderate elevations in transaminase levels, indicating impaired liver function and a potential link to hypolipidemia. However, the exact mechanism by which SARS-CoV-2-induced liver damage affects the biosynthesis of LDL-C remains to be established. Recent evidence from COVID-19 patients indicates a significant increase in IL-6 levels in 96% of those investigated, suggesting that proinflammatory cytokines and acute inflammation may play a crucial role in the disturbed lipid metabolism observed in COVID-19 patients [50]. Viral-induced inflammation can lead to dyslipidemia, specifically decreased LDL-C levels. The reduced LDL-C levels are likely not primarily caused by liver damage but rather influenced by acute inflammation and elevated levels of proinflammatory cytokines such as IL-6. These results demonstrate the importance of inflammation in altering lipid metabolism in COVID-19 infection. Upon admission to the hospital, COVID-19 patients experience a reduction in LDL-C levels, which persists throughout the course of treatment. However, upon discharge, LDL-C levels gradually return to their pre-infection levels. Total cholesterol follows a similar pattern, while HDL-C levels decrease initially and remain low even after recovery. Non-surviving COVID-19 patients experience a continuous decline in LDL-C, HDL-C, and total cholesterol levels until death. Critically ill patients also exhibit reduced HDL-C levels, while even mildly symptomatic patients can develop hypolipidemia that corresponds to disease severity [51]. LDL-C cholesterol shows promise as a potential prognostic indicator for poor outcomes in COVID-19 [52]. Infection with SARS-CoV-2 can also affect thyroid function, with mild reductions in TSH and FT4 levels observed in some COVID-19 patients [53]. The thyroid gland expresses higher levels of ACE2 and TMPRSS2 than the respiratory system, which may contribute to a variety of immune responses and manifestations. Moreover, the cytokine storm associated with COVID-19 may indirectly cause prolonged inflammation in the thyroid gland [50].

Drugs commonly used to treat COVID-19, such as corticosteroids or antiviral agents, can worsen hyperglycaemia, leading to lipodystrophy and insulin resistance [37]. An observational study revealed that hospitalized patients with COVID-19 who had hyperglycaemia demonstrated elevated levels of IL-6 and D-dimer, two markers associated with inflammation and a procoagulant state. Effective glucose control significantly decreased these levels, suggesting that hyperglycaemia contributes to heightened inflammation and a procoagulant state independently of viral factors. Therefore, both type 1 and type 2 diabetes mellitus (T1DM and T2DM), particularly when accompanied by inadequate glycemic control, present high-risk pre-existing conditions for various bacterial and viral infections, including SARS-CoV-2 [3,54]. The available research had mainly focused on patients with type 2 diabetes (T2DM) or lacked information on diabetes type, leaving uncertainty regarding the elevated risk of severe COVID-19 in type 1 diabetes (T1DM) patients. Recent evidence suggests, however, that individuals with type 1 diabetes mellitus (T1DM) are at an increased risk of experiencing severe outcomes related to COVID-19, such as mortality, ICU admission, and hospitalisation. Compared to non-diabetic individuals, T1DM patients experience a 3.5-fold increase in in-hospital deaths due to COVID-19, according to a study of the entire English population [55]. Individuals with T1DM and COVID-19 who had an HbA1c level greater than 10.0% (86 mmol/mol) were found to have significantly higher odds of mortality compared to those with an HbA1c level of 6.5–7.0% (48–53 mmol/mol). Similarly, a nationwide population-based study in Scotland showed that T1DM patients faced higher risks of COVID-19-related mortality and ICU admission compared to T2DM patients [56]. Finally, a recent prospective cohort study conducted in the USA confirmed these findings and further showed that T1DM patients had a higher risk of hospitalization for COVID-19 than T2DM patients [57]. It should not be neglected that SARS-CoV-2 patients with diabetes are more prone to the development of severe clinical presentation [58,59], as well as the complicated course of the disease, especially when they are not regulated properly. A large prospective cohort study that included 9058 patients in Romania showed that patients with T2DM were associated with higher intensive care unit mortality [60]. Those results are also supported by meta-analysis, including data from 6,653,207 patients associating diabetes mellitus with both hospital and community-based mortality and risk for developing severe clinical presentation and need for hospitalization [61]. Furthermore, SARS-CoV-2 has been identified as a plausible culprit for the onset of diabetes mellitus in previously healthy individuals [62,63]. Moreover, there is a correlation between COVID-19 and notable impairment in metabolic function in both new-onset and pre-existing diabetes cases, potentially leading to severe conditions like diabetic ketoacidosis (DKA) and hyperglycaemic hyperosmolar state (HHS) [8,64,65,66].

## 4. COVID-19-Induced Diabetes

The association between diabetes and COVID-19 is probably bidirectional. Diabetes is known as a serious disease and comorbidity associated with a more severe clinical presentation and worse prognosis in many infectious and other diseases [67,68,69,70,71,72,73,73]. New-onset hyperglycaemia has been increasingly observed in adults with no history of diabetes in association with COVID-19, accompanied with significant morbidity and mortality. Although infection-induced inflammation and cytokine activation leading to insulin resistance may cause stress hyperglycaemia, it is unknown to what extent the direct destruction of islet cells by the virus, resulting in decreased insulin production and release, contributes [9]. Interestingly, COVID-19 infection has been linked to the development of diabetes, evident through sudden onset hyperglycaemia in non-diabetic individuals, diabetic ketoacidosis in pre-existing diabetic patients with COVID-19, and the emergence of diabetes in patients with COVID-19 [13,74,75,76], shown in Table 1.

Like other viruses, SARS-CoV-2 infections can trigger a stress response that may decrease insulin secretion, activate the release of cortisol and adrenaline, and stimulate excessive gluconeogenesis, leading to temporary hyperglycaemia. These mechanisms do not inevitably result in diabetes [80]. COVID-19 infection has been associated with a distinctive range of newly developed diabetes variations, including some that appear to be unique to the disease. While most large-scale studies have categorized new-onset diabetes as either type 1 or type 2, recent case reports have suggested that COVID-induced diabetes can take on different forms. Omotosho et al. presented a case study of a woman who developed latent autoimmune diabetes of adulthood (LADA) following a COVID-19 infection [81]. Positive tests for islet cell and glutamic acid decarboxylase (GAD) antibodies confirmed the patient’s type 1 diabetes diagnosis. Marchand et al. also reported a case of LADA in a patient infected with COVID-19 [82].

A random effects meta-analysis determined that the overall incidence of new-onset diabetes among COVID-19 patients was 14.4% [62]. Additionally, a systematic review and meta-analysis of eight cohort studies, which included over forty-seven million individuals, demonstrated that COVID-19 was associated with a 66% increase in the risk of diabetes compared to those who did not contract COVID-19. The risk was not influenced by variables such as age, gender, or study quality [83]. Patients with newly diagnosed diabetes as a result of COVID-19 have a greater risk of hospitalization and death compared to those who are normoglycaemic or have only temporary hyperglycaemia. These patients with pre-existing or new-onset diabetes associated with COVID-19 also have more severe complications, such as acute respiratory distress syndrome, acute renal failure, shock, and low albumin levels, compared to those with normal or temporarily elevated blood sugar levels [11]. Furthermore, patients with COVID-19 are also more prone to ketoacidosis [84,85,86], which could indicate the diabetogenic potential of SARS-CoV-2. The mechanisms mentioned were proposed by Sathish et al. [87] and some other authors [88,89]. Some studies showed that diabetes is related to prolonged hospitalization of patients with COVID-19 [90] and with worse disease outcomes [27,28,79,91,92,93,94,95,96]. It is also concluded that patients with diabetes mellitus are more prone to developing severe symptoms of COVID-19 [28,93,94,96]. Moreover, newly diagnosed diabetes during SARS-CoV-2 infection has been linked to an even worse prognosis than pre-existing, probably due to insufficient diabetes regulation [62,92,93,97].

A previous study that involved more than 180,000 veterans showed that individuals who had recovered from COVID-19 were 40% more likely to develop diabetes than those who had not previously been diagnosed with COVID-19. As mentioned, another study revealed that as much as 14% of individuals who were hospitalized for COVID-19 were subsequently diagnosed with diabetes [83]. Another meta-analysis that included 4,270,747 SARS-CoV-2 positive patients surviving the disease and 43,203,759 control patients demonstrated a higher risk of diagnosing diabetes in recovered COVID-19 patients than in the control group [83]. Those data could be in favour of the diabetogenic potential of SARS-CoV-2 or could have a connection with corticosteroid treatment, which can worsen hyperglycaemia, resulting in a negative impact on patients’ physiological processes [28,98]. However, the SARS-CoV-2 pandemic influenced diabetic patients in other ways, such as health availability and support, which were mostly insufficient, at least at the beginning of the pandemic [99,100,101,102].

### 4.1. Type 1 Diabetes Mellitus (T1DM)

Although type 1 diabetes is autoimmune in nature, its onset usually necessitates an environmental trigger, such as an infection [103]. In the case of SARS-CoV-2, it is suggested that direct infection, coupled with the inflammatory response and interactions with the renin–angiotensin system, can lead to damage to pancreatic cells and the development of new-onset diabetes. Case reports of individuals with recent SARS-CoV-2 infection presenting with new-onset T1DM and DKA suggest that SARS-CoV-2 infection may expedite the development of T1DM or elevate the susceptibility to its metabolic complications [66,77,78,104]. There is much speculation surrounding the suggestion that exposure to SARS-CoV-2 could have triggered the onset of T1DM, which may have contributed to the rise in DKA. However, there is insufficient evidence to confirm whether this new-onset diabetes represents classic T1DM or a distinct form of diabetes. It remains unclear whether the severe COVID-19-induced hyperglycaemia observed in some individuals would resolve over time, as was observed with SARS-CoV-1-induced diabetes [1]. The precise mechanisms by which SARS-CoV-2 increases the risk of T1DM are still being investigated. However, it is known that the destruction of β-cells can initiate the spread of epitopes, leading to the activation of CD-8 T cells and the production of a broader spectrum of autoantibodies that target various islet cells, such as insulin, glutamic acid decarboxylase, and protein tyrosine phosphatase. This autoimmune response depletes functional β-cells, resulting in hyperglycemia and the clinical manifestation of type 1 diabetes [105].

### 4.2. Type 2 Diabetes Mellitus (T2DM)

Studies have indicated that acute COVID-19 more often may exacerbate pre-existing prediabetes or T2DM [106]. Individuals who are hospitalized for acute COVID-19 infections may have undetected diabetes mellitus. The pandemic-related changes in lifestyle, such as decreased physical activity due to measures like lockdowns, may have played a role in the increased weight gain and glyco-metabolic syndrome observed in people with prediabetes. Such changes may also raise the risk of developing new-onset diabetes in the post-infectious stage [79]. COVID-19 can elevate stress hormones, such as adrenaline and cortisol, which may trigger the production of glucose, resulting in hyperglycaemia [107]. Also, direct cytotoxic injury to pancreatic cells caused by SARS-CoV-2 infection may result in reduced insulin production [108]. COVID-19 may exacerbate pre-existing T2DM or prediabetes. Certain studies suggest that these conditions are transient and may resolve with time, but this hypothesis requires ongoing investigation in the future [106].

### 4.3. COVID-19-Vaccine-Induced Diabetes Mellitus

There are speculations about the impact of COVID-19 vaccines on diabetes mellitus development. For now, there are mostly case reports for such events; patients are usually presenting with ketoacidosis [109,110,111], hyperglycaemia [112,113,114], or hyperosmolar state [113]. There were some observations that vaccines could precipitate hyperglycaemia and other complications in patients that already have diabetes [115], but a study conducted on 350,936 cases did not find such a link [116], as well as a study completed in paediatric patients [117]. Furthermore, there are reports about more frequent adverse reactions to the vaccines with diabetic patients [118,119], but other researchers did not find such a relation [58]. There were concerns that, since diabetes mellitus is a procoagulatory state, vaccines could precipitate thromboembolic incidents, but a study investigating coagulation pathways in T1DM and T2DM patients after vaccination did not find significant differences compared to healthy individuals [59]. Vaccines not only reduce the chance of severe clinical presentation and hospital admission in diabetic patients [120] but could also have a protective effect and reduce the possibility of developing diabetes mellitus after COVID-19 in healthy individuals [108,121]. Nevertheless, patients with diabetes mellitus have decreased antibody and memory β-cell response to the vaccine [58,122,123], and there are reports that vaccines could be less effective in those individuals [124,125].

## 5. Underlying Pathophysiological Mechanism of COVID-19-Induced Diabetes Mellitus

**Figure 1:**
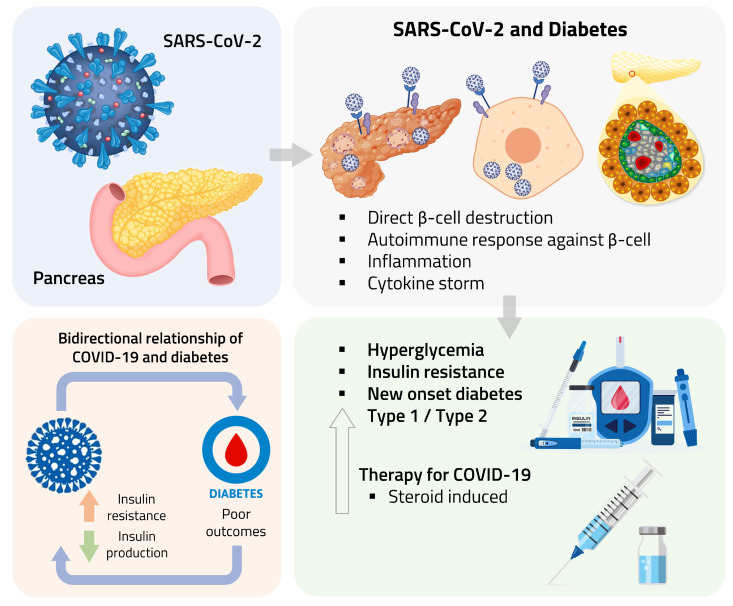
Potential pathophysiological mechanisms of COVID-19-induced diabetes.

The pathogenesis of SARS-CoV-2-induced new-onset diabetes is complex and not yet fully understood. As previously described, it may involve direct β-cell damage, systemic-inflammation-induced insulin resistance, and hormonal dysregulation. Additionally, treatment with glucocorticoids for COVID-19 may increase the risk of new-onset diabetes due to their association with hyperglycaemia and insulin resistance [126,127], shown in Figure 1.

### 5.1. Direct and Indirect β-Cell Damage

The onset of acute hyperglycaemia during coronavirus infection has been linked to the binding of the virus to the ACE2 receptor located in pancreatic islet cells [128]. The SARS-CoV-2 virus infects cells by attaching to receptors such as ACE2, TMPRSS2, and DPP-4, which are present not only in alveolar cells but also in cells of the pancreas, heart, and small intestine. The expression of ACE2 has been found to be more prominent in the pancreas than in the lungs and has been detected in both the exocrine glands and islets of the pancreas, including β-cells [129]. Autopsies of COVID-19 patients have confirmed the presence of the virus in β-cells and the potential for replication within the endocrine pancreas. The virus infects pancreatic β-cells and causes transdifferentiation, leading to decreased insulin secretion and increased production of glucagon and trypsin 1 [130]. The findings of Muller et al. indicate that SARS-CoV-2 infection of β-cells can result in hormone-negative cells, supporting the theory that the disruption of glucose regulation observed in COVID-19 patients may play a role in the development of new-onset diabetes [131]. The pro-inflammatory cytokines and acute-phase reactants triggered by COVID-19 may directly induce inflammation and harm pancreatic β-cells [132]. Individuals with acute SARS-CoV-2 infection may experience a cytokine storm, an intensely inflammatory state affecting various organs in the body, including the pancreas. This can give rise to acute pancreatitis and degeneration of the pancreatic islet cells, potentially resulting in hyperglycaemia and diabetes if the damage is extensive [133].

#### 5.1.1. Indirect β-Cell Damage: Autoimmunity and Inflammation

COVID-19 has been linked to the development of various autoimmune disorders, including systemic lupus erythematosus (SLE), Guillain–Barré syndrome, and Grave’s disease. The autoimmunity of β-cells could be explained by molecular mimicry [134]. Molecular mimicry refers to a phenomenon where a viral epitope shares similarities with a host islet protein, potentially triggering an autoimmune response against the host tissue in susceptible individuals. However, studies conducted to investigate molecular mimicry have produced inconclusive findings. It is likely that molecular mimicry does not initiate the autoimmune process independently but rather accelerates it once it has already been initiated [135]. Prolonged infection of β-cells leads to the continual overexpression of MHC-1, which in turn leads to the persistent presentation of β-cells epitopes to the immune system. This sustained presentation of antigens contributes to the promotion of autoimmunity [64]. Excessive formation of neutrophil extracellular traps (NETs) during COVID-19 infection may also play a role in autoimmunity. NETs are complex structures composed of DNA, histones, microbicidal proteins, and oxidant enzymes that neutrophils release to contain infections. While neutrophil extracellular traps (NETs) play a crucial role in preventing the invasion of pathogens, their uncontrolled formation can have detrimental effects, including the development of autoimmune inflammation and tissue damage [136]. The ACE2 receptor plays a pivotal role in anti-inflammatory pathways by producing angiotensin 1–7, which has vasodilatory and antifibrotic effects, by converting angiotensin II into inactive angiotensin 1–7. In SARS-CoV-2 infection, ACE2 expression is reduced, resulting in reduced levels of angiotensin 1–7 and increased inflammation and coagulability [137]. Chee et al. have proposed a hypothesis stating that reduced levels of ACE2 and the detrimental effects of angiotensin II cause diminished blood flow to pancreatic β-cells, leading to β-cell dysfunction and glycaemic dysregulation. Angiotensin II also has a pro-inflammatory effect, increasing macrophage and monocyte infiltration, further exacerbating the disruption of β-cell function. These mechanisms are likely to cause β-cell function disruptions, resulting in glycaemic dysregulation [104,138].

#### 5.1.2. Insulin Resistance

Patients with COVID-19 often experience a decreased sensitivity to insulin due to the damage to their β-cells and may require an increase in insulin dosage, especially during feverish episodes [139]. According to research by He et al., COVID-19 was found to cause new-onset insulin resistance in patients without a prior history of metabolic disease [140]. The research demonstrated a reduction in the activity of the REST transcription factor, which is connected to alterations in the expression of essential genes involved in glucose and lipid metabolism. Additionally, an increase in propionic and isobutyric acids was noted, and previous animal studies have established a link between these acids and insulin resistance. Interestingly, the BMI range for subjects with COVID-19 in this study was between 20.5 and 24.6, suggesting that lean individuals may also develop insulin resistance, independent of traditional risk factors such as high BMI. This study also showed that insulin resistance persists even after SARS-CoV-2 has been eliminated, suggesting potential long-term consequences for COVID-19 patients [140].

The induction of the integrated stress response (ISR) due to stressors can activate a group of four serine/threonine kinases, including RNA-dependent protein kinases that may phosphorylate insulin receptor substrates (IRS) at serine, which can suppress the insulin signalling pathway. In the case of SARS-CoV-2 infection, viral RNA fragments may activate kinase and induce insulin resistance [137]. Additionally, cytokine storm, a condition characterized by high levels of pro-inflammatory cytokines, is known to activate the serine/threonine kinase family associated with the ISR, resulting in insulin resistance [141,142]. In a study by Šestan et al., viral-infection-induced production of interferon gamma (IFN) reduced insulin receptors in skeletal muscle, resulting in insulin resistance. This mechanism could contribute to the insulin resistance observed in patients with COVID-19-induced diabetes, both adults and children [143,144,145]. Cellular stress during acute inflammation may stimulate accelerated lipolysis, leading to increased levels of free fatty acids in circulation and relative insulin deficiency [132]. Once more, the potential impact of glucocorticoid therapy on precipitating insulin resistance should not be overlooked.

## 6. Future Directions and Conclusions

The study of COVID-19’s impact on glucose regulation is a pressing area of research. The underlying cause of glucose imbalance in COVID-19 patients is complex and multi-dimensional, encompassing insulin resistance and β-cell dysfunction. Clinical data have shown that COVID-19 patients require increased insulin doses to maintain glucose control and exhibit significant fluctuations in glucose levels. Scientists are exploring SARS-CoV-2’s mechanisms of β-cell destruction through its known receptors, such as ACE2, and other potential entry points that require further investigation. Additionally, the connection between the β-cells and the endothelium, which is crucial for intact β-cell function, may also contribute to β-cell dysfunction indirectly. Timely recognition and management of patients with new-onset diabetes post-COVID-19 is of paramount importance as it has been linked to unfavourable outcomes. Further research is imperative to fully understand this novel type of diabetes and to establish effective management strategies. The long-term effects of COVID-19 on glucose metabolism are still unclear, including whether these disruptions are permanent or if the virus can cause a novel form of diabetes. Further research is imperative to fully elucidate this novel type of diabetes and to establish effective management strategies and potential treatment options.

## Figures and Tables

**Table 1:** Studies that have described new-onset diabetes in COVID-19 patients.

Authors	Type of Study	Country	Number of Cases	Results
Sathish et al. [62]	Meta-analysis	China, Italy, US	3711 patients	14.4% of the population had new-onset diabetes
Sathish et al. [63]	Retrospective study	India	102 patients	20.6% of the population had new-onset diabetes
Li et al. [11]	Retrospective study	China	453 patients	20.8% of the population had new-onset diabetes
Unsworth et al. [65]	Retrospective study	UK	30 children	80% increase in new-onset T1DM
Tittel et al. [77]	Prospective study	Germany	Children from 216 paediatric diabetes centres	T1DM incidence increased from 16.4 per 100,000 to 22.2 per 100,000
Salmi et al. [78]	Retrospective study	Finland	Children admitted to PICU due to new-onset diabetes compared with the pre-pandemic period	The number of children admitted to PICU due to new-onset diabetes increased from 6.25 in 2016 to 20 in 2020.
Montefusco et al. [75]	Retrospective study	Italy	551 patients	46% were hyperglycaemic
Kamrath et al. [66]	Prospective study	Germany	532 newly diagnosed T1DM	The frequency of diabetic ketoacidosis was 44.7%
Fadini et al. [13]	Retrospective study	Italy	413 patients	5% had new-onset diabetes
Ghosh et al. [76]	Retrospective study	India	555 patients with new-onset diabetes	Patients with new-onset diabetes had worse glycaemic parameters
Shrestha et al. [79]	Meta-analysis	US, China, France, India, Italy	1943 patients across seven studies	The mortality rate in COVID-19-associated diabetes patients was 25%

## Abbreviations

The following abbreviations are used in this manuscript (in alphabetical order):

ACE2	Angiotensin-Converting Enzyme 2
BMI	Body Mass Index
COVID-19	Coronavirus Disease 2019
DKA	Diabetic Ketoacidosis
DNA	Deoxyribonucleic Acid
DPP-4	Dipeptidyl-Peptidase 4
FT4	Free Thyroxine
GAD	Glutamic Acid Decarboxylase
HbA1c	Hemoglobin A1c
HDL-C	High-Density Lipoprotein Cholesterol
HHS	Hyperglycaemic Hyperosmolar State
ICU	Intensive Care Unit
IFN	Interferon Gamma
IL-6	Interleukin 6
IRS	Insulin Receptor Substrates
ISR	Integrated Stress Response
LDL-C	Low-Density Lipoprotein Cholesterol
NET	Neutrophil Extracellular Trap
REST	Repressor Element-1 Silencing Transcription factor
RNA	Ribonucleic Acid
SARS-CoV-2	Severe Acute Respiratory Syndrome Coronavirus 2
SLE	Systemic Lupus Erythematosus
T1DM	Type 1 Diabetes Mellitus
T2DM	Type 2 Diabetes Mellitus
TMPRSS2	Transmembrane Serine Protease 2
TNF-alpha	Tumor Necrosis Factor Alpha
TSH	Thyroid-stimulating hormone
